# CXCL12 enhances angiogenesis through CXCR7 activation in human umbilical vein endothelial cells

**DOI:** 10.1038/s41598-017-08840-y

**Published:** 2017-08-15

**Authors:** Min Zhang, Lisha Qiu, Yanyan Zhang, Dongsheng Xu, Jialin C. Zheng, Li Jiang

**Affiliations:** 1 Division of Cardiology, Tongren Hospital, Shanghai Jiao Tong University School of Medicine, 1111 Xianxia Road, Shanghai, 200336 P.R. China; 2 Center for Neuroimmunology and Regenerative Therapy, Shanghai Tenth People’s Hospital, Tongji University School of Medicine, Shanghai, 20072 P.R. China; 3 Department of Pharmacology and Experimental Neurosciences, University of Nebraska Medical Center, 68198-5930, Nebraska Medical Center, Omaha, Nebraska United States; 4 State Key Laboratory of Virology, College of Life Sciences, Wuhan University, Wuhan, 430072 P.R. China; 5 Collaborative Innovation Center for Brain Science, Tongji University, Shanghai, 20072 P.R. China; 6 Department of Spine Surgery and Neural Rehabilitation Center, Tongji Hospital, Tongji University, Shanghai, 20065 P.R. China

## Abstract

Angiogenesis is the process by which new vessels form from existing vascular networks. Human umbilical vein endothelial cells (HUVECs) may contribute to the study of vascular repair and angiogenesis. The chemokine CXCL12 regulates multiple cell functions, including angiogenesis, mainly through its receptor CXCR4. In contrast to CXCL12/CXCR4, few studies have described roles for CXCR7 in vascular biology, and the downstream mechanism of CXCR7 in angiogenesis remains unclear. The results of the present study showed that CXCL12 dose-dependently enhanced angiogenesis in chorioallantoic membranes (CAMs) and HUVECs. The specific activation of CXCR7 with TC14012 (a CXCR7 agonist) resulted in the significant induction of tube formation in HUVECs and *in vivo*. Further evidence suggested that CXCL12 induced directional polarization and migration in the HUVECs, which is necessary for tube formation. Moreover, CXCR7 translocalization was observed during the polarization of HUVECs in stripe assays. Finally, treatment with TC14012 also significantly increased PI3K/Akt phosphorylation, and tube formation was blocked by treating HUVECs with an Akt inhibitor. Overall, this study indicated that CXCL12-stimulated CXCR7 acts as a functional receptor to activate Akt for angiogenesis in HUVECs and that CXCR7 may be a potential target molecule for endothelial regeneration and repair after vascular injury.

## Introduction

Many reports have revealed that angiogenesis is a compensatory and protective response to ischemic diseases. Thus, the promotion of angiogenesis is being investigated as a therapy for patients with ischemic diseases^1, 2^. Angiogenesis involves the proliferation and migration of endothelial cells, which are then reorganized into tubular formations to form vessel networks^3^. The chemokine CXCL12 (also called stromal cell derived factor 1α, SDF-1α) is a key factor in angiogenesis^4^, and it influences important physiological processes such as inflammation, wound healing and embryonic development^5, 6^. Findings from previous studies have demonstrated that autocrine CXCL12 can induce migration and tubulogenesis of human umbilical vein endothelial cells (HUVECs) and human microvascular endothelial cells (HMVECs)^7–9^, independently of VEGF, through the CXCR4 receptor^10–13^. For many years, CXCR4 has been considered a unique receptor of CXCL12 and the only mediator of CXCL12-induced biological effects^14, 15^. Recently, CXCR7 has been identified as a new atypical G protein-coupled receptor for CXCL12^16^. Previous work has shown that CXCR7 is expressed in human endothelial cells, including HUVECs and HMVECs, pulmonary microvascular endothelial cells, and endothelial cells within the central nervous system^17–21^. Although endothelial cells (ECs) express very low levels of CXCR7 under normal physiological conditions *in vivo* and normal culture conditions *in vitro*, CXCR7 expression is induced in activated EC. This has suggested that CXCR7 plays a role in EC during physiological stress. Recently, some reports have demonstrated that the levels of CXCR7 expression were increased in a variety of cancer tissues^22–27^. Further, CXCR7 has been shown to play a key role in tumor endothelial cell (TEC) angiogenesis, and its inhibition decreased angiogenesis in TECs^28^. These findings suggest that CXCR7 may participate in tumor angiogenesis^29^. Nevertheless, there are still few reports addressing the effects of CXCR7 in angiogenesis of ECs *in vitro* and in animal models *in vivo*.

The Akt signaling pathway plays a central role in regulating cellular functions, including proliferation, apoptosis, mobility, and angiogenesis^30–33^. Considering the fact that aberrant Akt signaling leads to impaired angiogenesis and vascular remodeling^34^, an important issue that requires further study is whether CXCR7 can activate Akt-mediated phosphorylation pathway, since it plays a role in angiogenesis.

Therefore, the present study aims to clarify the role of CXCR7 and the regulation of the PI3K/Akt pathway in neovascularization.

## Results

### CXCL12 enhanced angiogenesis in HUVECs

**Figure 1:**
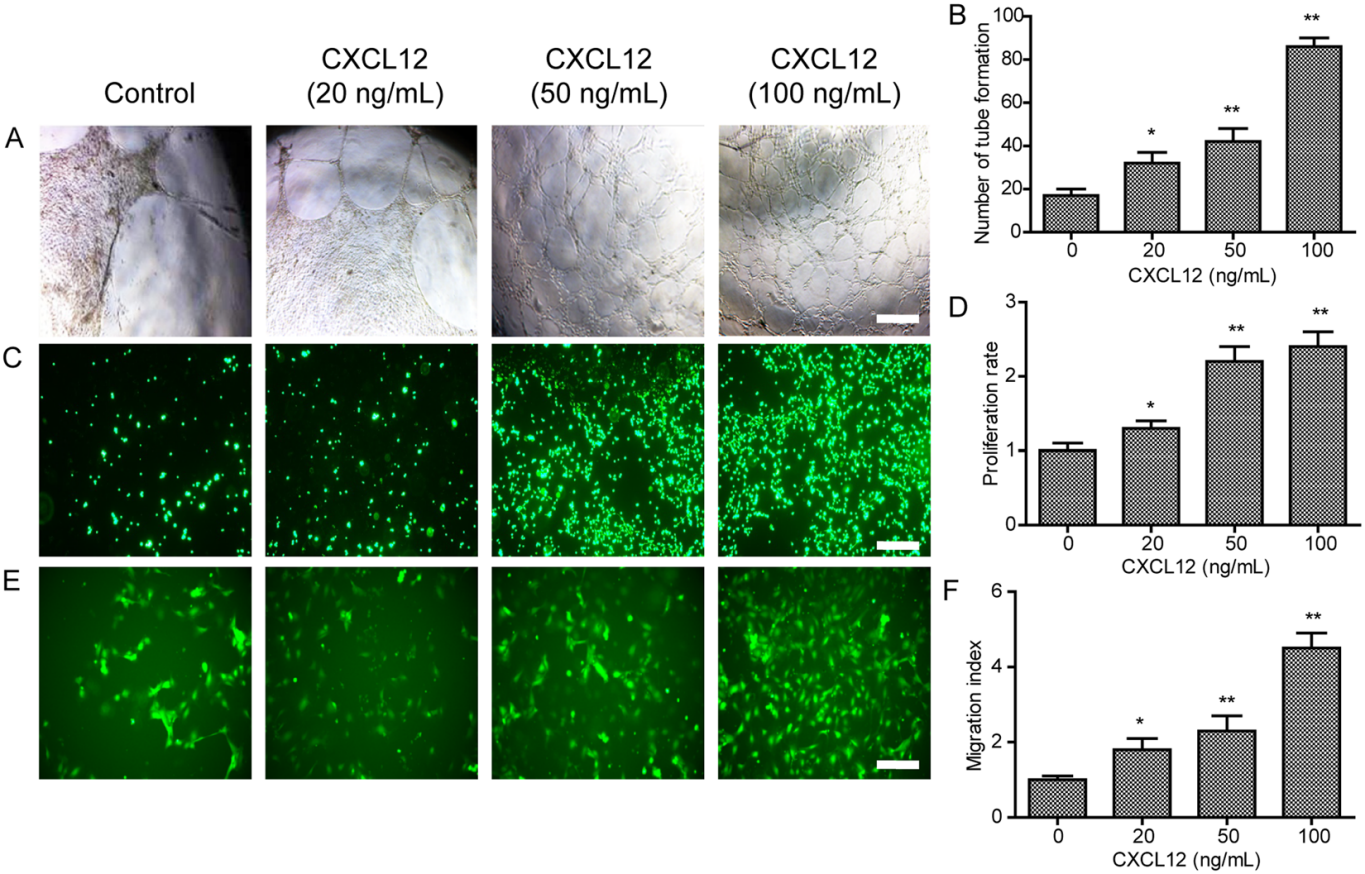
The CXCL12 enhances tube formation, proliferation and migration in HUVECs. (**A**) The tube formation of HUVECs increased with CXCL12 (0, 20, 50 and 100 ng/mL) treatment for 24 h in matrigel. (**B**) Quantitative analysis of the tube formation of HUVECs as shown in A. (**C**) The proliferation of HUVECs increased with CXCL12 (0, 20, 50 and 100 ng/mL) treatment for 72 h in culture. (**D**) Quantitative analysis of the proliferation of HUVECs as shown in C. (**E**) The migration of HUVECs increased with CXCL12 (0, 20, 50 and 100 ng/mL) treatment for 8 h in transwell. (**F**) Quantitative analysis of the migration of HUVECs as shown in E. Values are shown as mean ± SD, n = 3, *p < 0.05/**p < 0.01 versus control. Scale bars, 200 μm.

To test whether CXCL12 induced angiogenesis in HUVECs, we exposed the HUVECs to various doses of CXCL12 (0–100 ng/mL), and they showed dose-dependent enhancement of tube formation (Fig. 1A), proliferation (Fig. 1B) and migration (Fig. 1C) of the cells. These results revealed that CXCL12 induced angiogenesis in HUVECs *in vitro*.

### CXCL12 enhanced angiogenesis in CAM

**Figure 2:**
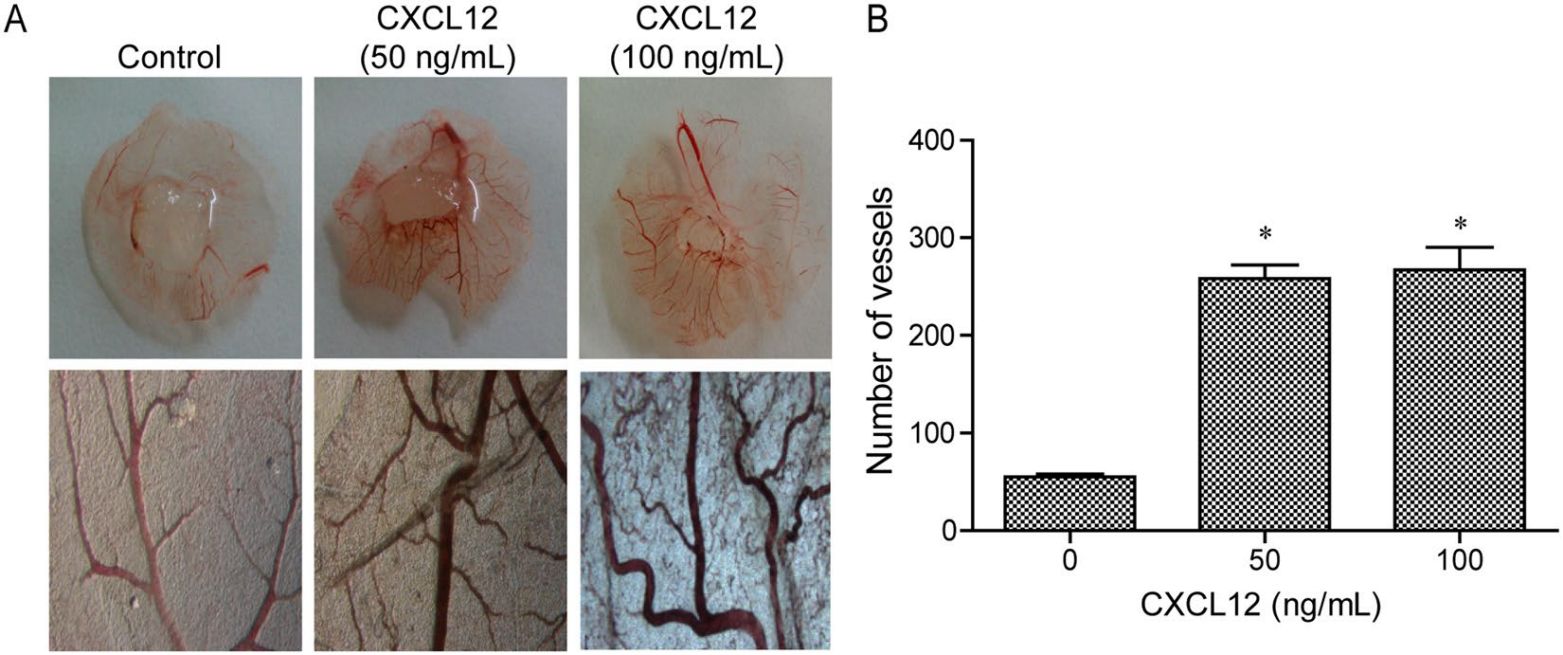
The CXCL12 enhances angiogenesis in chicken chorioallantoic membrane (CAM). New vessels increased with CXCL12 (0, 50 and 100 ng/mL) treatment in CAM. (**B**) Quantitative analysis of the new vessels as shown in A, mean ± SD from three independent experiments were used. *p < 0.05 versus control.

To investigate the effect of CXCL12 on angiogenesis, we used the CAM assay. In the CAM model, the CXCL12 group showed significantly more new blood vessels than the control group (Fig. 2A). Moreover, the quantitative data confirmed that CXCL12 in doses of 50 and 100 ng/mL induced neovessel formation (Fig. 2B, P < 0.05). Overall, the data suggested that CXCL12 enhanced the capacity for angiogenesis *in vivo*.

### The activation of CXCR7 promoted tube formation in HUVECs

**Figure 3:**
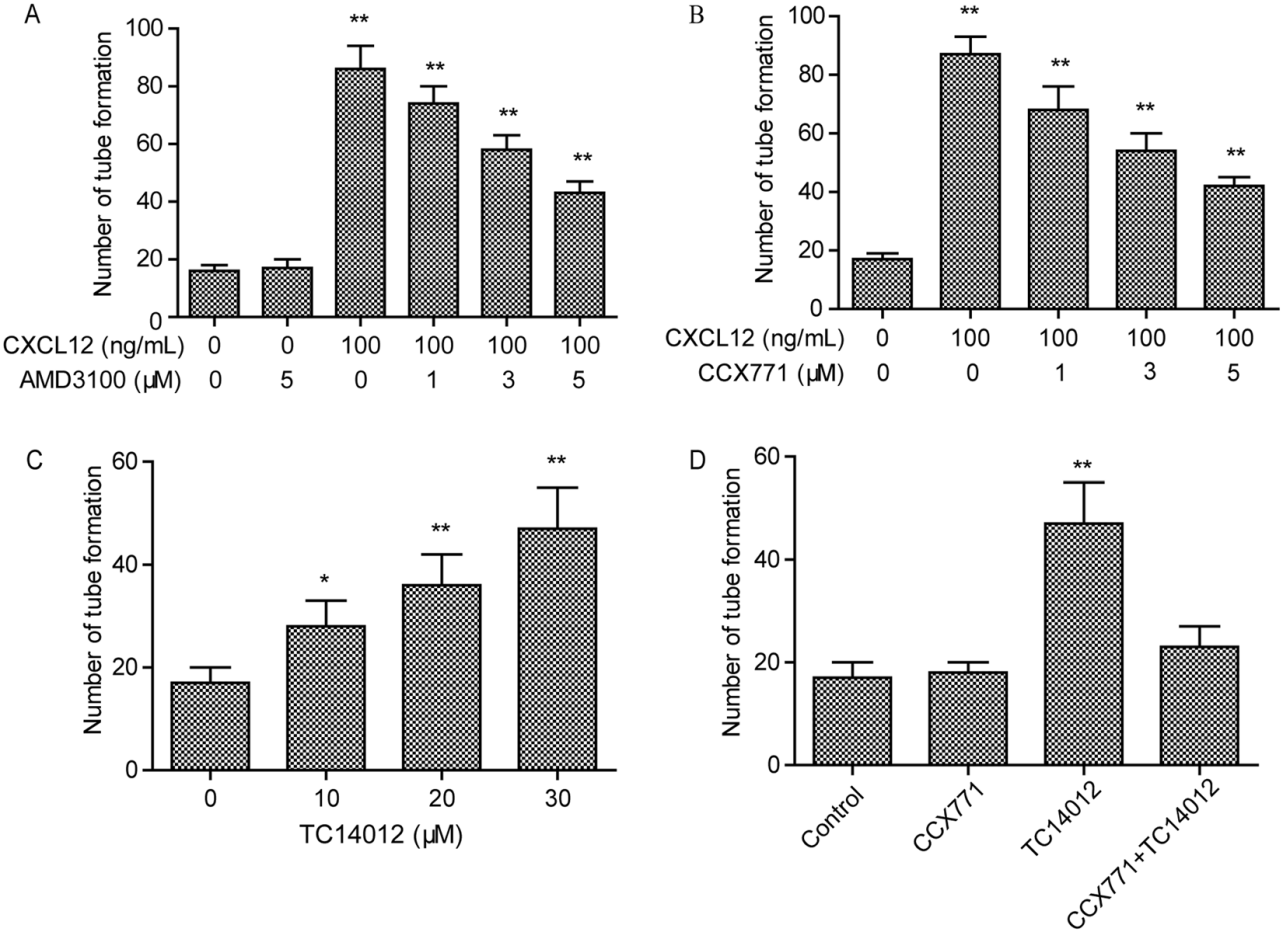
CXCL12 mediates tube formation through both CXCR4 and CXCR7 in HUVECs. (**A**) CXCR4 inhibitor AMD3100 (0–5 μM) does not completely block the tube formation mediates by CXCL12 in HUVECs. *p < 0.05/**p < 0.01 versus CXCL12. (**B**) CXCR7 inhibitor CCX771 (0–5 μM) does not completely block the tube formation mediates by CXCL12 in HUVECs. *p < 0.05/**p < 0.01 versus CXCL12. (**C**) CXCR7 special agonists TC14012 (0–30 μM) enhanced the tube formation in HUVECs. (**D**) CXCR7 inhibitor CCX771 (5 μM) completely blocked the tube formation mediates by TC14012 (30 μM) in HUVECs. *p < 0.05/**p < 0.01 versus CXCL12. **p < 0.01 versus control. Values are shown as mean ± SD, n = 3.

Tube formation in ECs is one of the critical steps in neovascularization. We investigated whether CXCR7 activation increased tube formation in HUVECs. To this end, we treated the HUVECs with the CXCR4 antagonist AMD3100 (Fig. 3A) or the CXCR7 antagonist CCX771 (Fig. 3B) with or without CXCL12 or the CXCR7 agonist TC14012 (Fig. 3C) and then measured tube formation. As shown in Fig. 3A, the CXCR4 antagonist AMD3100 caused a decrease in the number of tubes formed. Moreover, the CXCR7 antagonist CCX771 (Fig. 3B) also significantly inhibited tube formation. In HUVECs treated with TC14012 (Fig. 3C), the specific activation of CXCR7 could significantly promote tube formation in the HUVECs. This demonstrated that CCX771 (5 μM) did not affect tube formation when administered as a pretreatment in the HUVECs. In HUVECs treated with CCX771 (5 μM) and TC14012 (30 μM), CCX771 was able to inhibit the effects of TC14012 (Fig. 3D). These results suggest that CXCR7 regulates tube formation in HUVECs *in vitro*.

### CXCL12-induced directional cell polarization is dependent on CXCR7 and CXCR4

**Figure 4:**
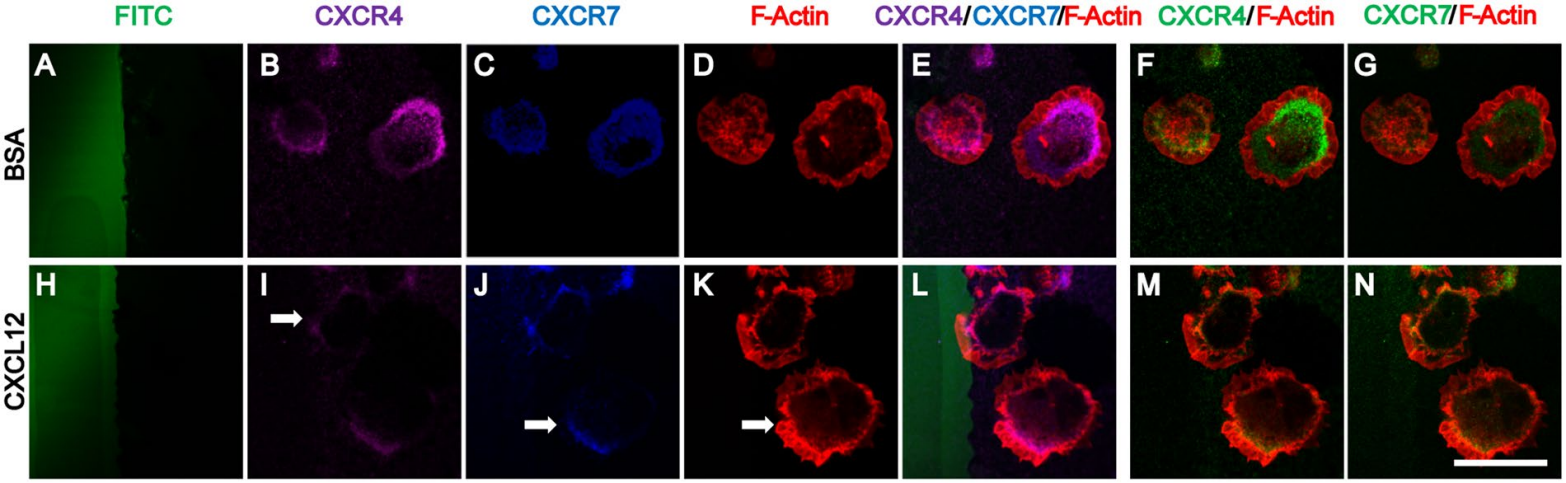
CXCL12 induces CXCR4/CXCR7 and cell polarization in HUVECs. HUVECs were cultured on plastic Petri dishes (pretreated with PDL) with BSA stripes (the control group) and BSA plus CXCL12 stripes (CXCL12 group) for 5 min. HUVECs were fixed and stained with antibodies against CXCR4 (purple), CXCR7 (blue) and F-Actin (red). The micro stripes of fluorescein-conjugated BSA (**A**) or CXCL12 (**H**) are shown. CXCR4 (**B**), CXCR7 (**C**) and F-Actin (**D**) in HUVECs cultured on BSA stripes stayed in the resting state, while the polarization of CXCR4 (arrow: **I**), CXCR7 (arrow: **J**) and F-Actin (arrow: **K**) was observed in HUVECs cultured on CXCL12 stripes for 5 min. Moreover, the HUVECs in the BSA control group showed no morphological changes (**E**–**G**). The CXCL12 group HUVECs shows polarization towards the CXCL12 stripe (**L**–**N**). Scale bar: 10 μm.

To assess whether CXCR7 and CXCR4 are involved in the polarization and migration of CXCL12-treated HUVECs, we performed stripe assays. Firstly, control and CXCL12 stripes were established by pre-coating fluorescein isothiocyanate (FITC)-BSA (Fig. 4A) and FITC-CXCL12 (Fig. 4H) protein on a polydimethylsiloxane (PDMS) stamp. The BSA-treated sample was used as the control. In the BSA control stripes, CXCR4 (Fig. 4B) and CXCR7 (Fig. 4C) were localized around the cytoplasm and the cell periphery. However, after exposure to the CXCL12 stripes for 5 min, CXCR4 (Fig. 4I) and CXCR7 (Fig. 4J) re-localized towards the CXCL12 stripes (indicated by arrows). Moreover, the lamellipodia had polarized towards the CXCL12 stripes after 5 min of treatment (Fig. 4K, pointed by arrows), suggesting that the cells were polarized for migration towards the CXCL12 stripes. In contrast, in the control group, HUVECs formed random filopodia and no lamellipodia towards BSA stripes (Fig. 4D), suggesting that cells were not polarized for migration. F-Actin, CXCR4 and CXCR7 were localized in the lamellipodia of polarized cells (Fig. 4L–N) in the CXCL12 group, suggesting their involvement in the cytoskeletal reorganization during cellular polarization and migration. In contrast, in the BSA control group, under resting conditions, F-Actin, CXCR4 and CXCR7 were distributed uniformly around the plasma membrane (Fig. 4E,F and G). All of the data suggested that CXCR7 and CXCR4 were involved in regulating cytoskeletal reorganization and polarization in HUVECs.

### The activation of CXCR7 stimulated angiogenesis *in vivo*

**Figure 5:**
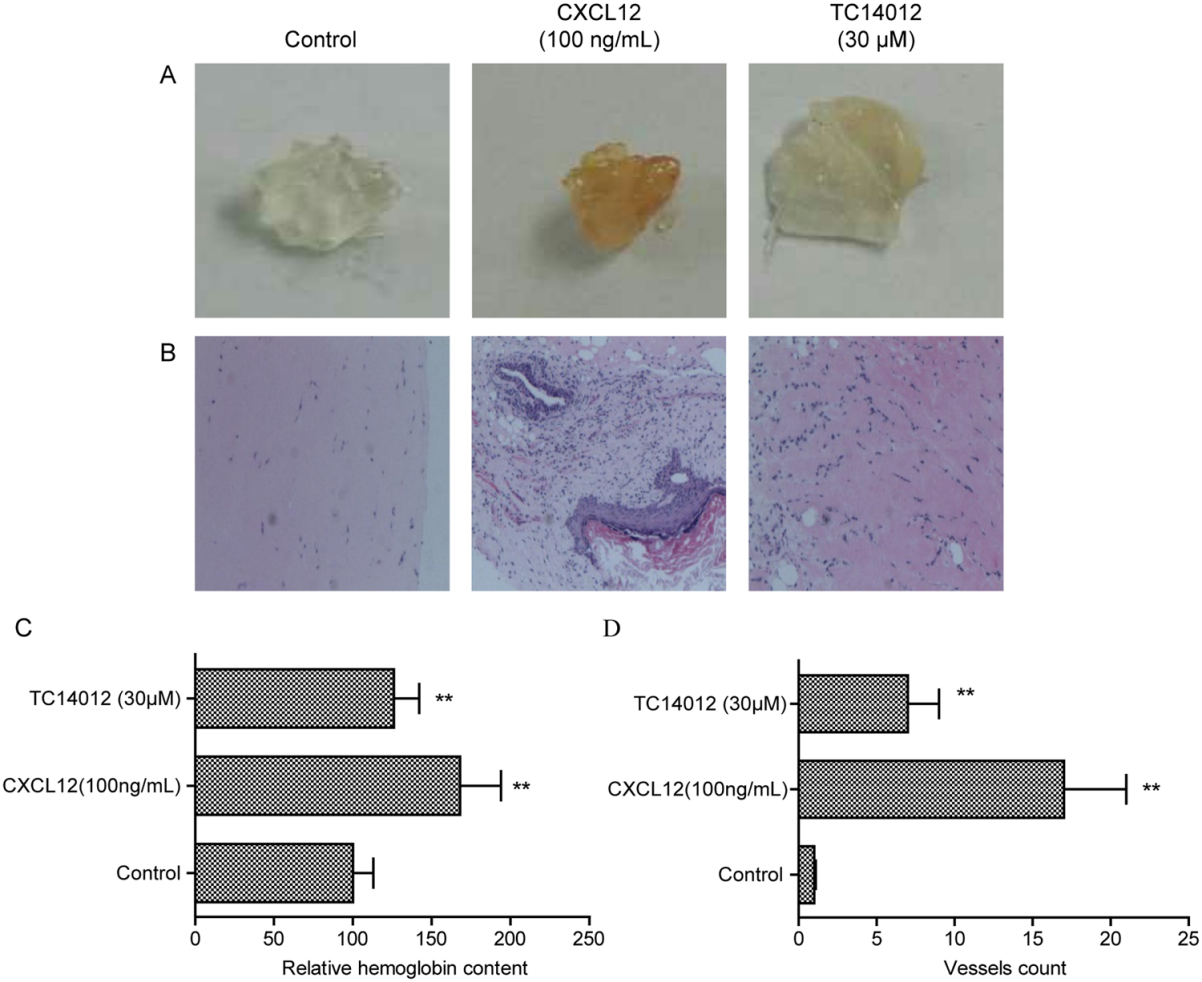
Activation of CXCR7 induces angiogenesis *in vivo*. (**A**) Mice were injected with Matrigel-containing CXCL12 (100 ng/mL) or TC14012 (30 μM) as indicted to induce angiogenesis *in vivo* for 10 days, Matrigel plugs were removed. (**B**) Representative HE staining of Matrigel plugs from control group, CXCL12 treated group and TC14012 treated group. Magnification 40×. (**C**) Quantification of hemoglobin content in the Matrigel plugs. (**D**) Quantification of the number of vessels in sections B. Values are shown as mean ± SD; *p < 0.05/**p < 0.01 versus control.

The effects of CXCL12 and TC14012 on new vessel formation and hemoglobin concentration in Matrigel plugs were investigated by subcutaneous injection of Matrigel into Balb/c mice. The Matrigel plugs that contained CXCL12 or TC14012 showed a grossly red appearance after 10 days (Fig. 5A). The H&E staining of paraffin-embedded Matrigel plug sections also revealed that new vessel formation was enhanced in CXCL12- or TC14012-stimulated plugs (Fig. 5B). Relative hemoglobin content as estimated by Drabkin’s method was also significantly increased in the CXCL12- or TC14012-treated Matrigel plugs in mice (Fig. 5C). Quantification of blood vessels (Fig. 5D) by H&E staining revealed that CXCL12 or TC14012 stimulated vessel growth as expected.

These data indicated that CXCL12 or TC14012 effectively enhanced tube formation by the ECs and angiogenesis in the Matrigel plugs. Interestingly, specifically activating CXCR7 by TC14012 could enhance angiogenesis *in vivo*.

### CXCR7 initiated tube formation via PI3K/Akt signaling

**Figure 6:**
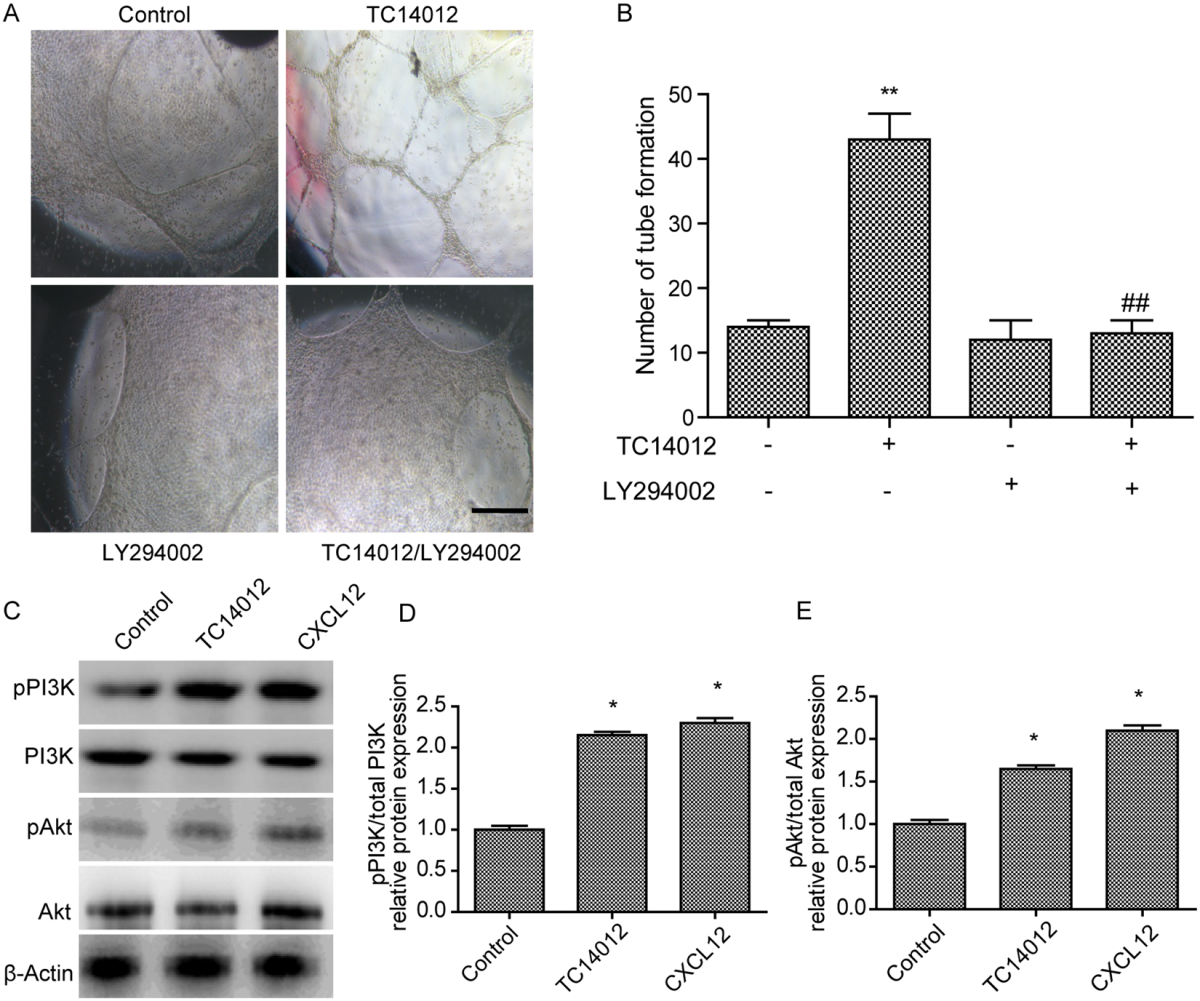
Tube formation mediates by TC14012 is blocked by PI3K/Akt inhibitor LY294002 in HUVECs. (**A**) LY294002 (10 μM) inhibited tube formation of HUVECs induced by TC14012 (30 μM). (**B**) Quantitative analysis of the tube formation of HUVECs as shown in A. (**C**) Western blots for PI3K/Akt of HUVECs exposed to TC14012 (30 μM) or CXCL12 (100 ng/mL). Representative WB showed that TC14012 (30 μM) or CXCL12 (100 ng/mL) induced protein expression of pPI3K (**D**) and pAkt (**E**) expression in HUVECs. Results are given as mean ± SD of three independent experiments. **p < 0.01 versus control, ^##^p < 0.01 versus TC14012.

We also evaluated whether the PI3K/Akt signaling pathway was required for CXCR7-initiated tube formation in HUVECs. Pretreatment with LY294002 (PI3K inhibitor, 10 μM) for 30 min significantly attenuated TC14012-induced tube formation in the HUVECs. Treatment with LY294002 alone did not change tube formation (Fig. 6A and B). These data suggested that the PI3K/Akt signaling pathway was necessary for CXCR7-initiated tube formation in the HUVECs.

As CXCR7 activation was shown to enhance tube formation in the HUVECs, the signaling pathways involved in this mechanism were investigated. CXCL12 or TC14012 significantly induced the phosphorylation of PI3K and Akt in the HUVECs (*p* < 0.05, Fig. 6C). The levels of total PI3K and Akt did not change, but the levels of pPI3K (Fig. 6D) and pAkt (Fig. 6E) were increased in the CXCL12- or TC14012-treated HUVECs, and the subsequent signaling cascade enhanced tube formation in the HUVECs.

## Discussion

Previous investigations have provided evidence for the induction of neovascularization by CXCL12 in various *in vivo* and *in vitro* models^4, 35–37^. However, only a few reports have specifically focused on the potential angiogenic roles of CXCR7^38, 39^. Here, we found that CXCR7 signaling can at least contribute to the CXCL12-mediated promotion of angiogenesis. However, investigation of the role of CXCR7 in the migration of ECs has been largely neglected so far. The results from the present study provide the first evidence for CXCL12-induced CXCR7 polarization of HUVECs as a key event in its angiogenic effect on the ECs. Moreover, our study also demonstrated that the blockade of CXCR7 resulted in the down-regulation of the Akt signaling pathway, which implied that the Akt pathway is involved in CXCR7-mediated angiogenesis in HUVECs.

Many previous investigations mainly focused on the effects of the CXCL12-CXCR4 axis on proliferation^13^, apoptosis^40^, migration^41^ and tube formation in the endothelial cells^42^, as well as the related signal transduction pathways, including MAPK, NF-κB and Akt^43–45^. To determine the role of CXCR4, AMD3100^46^, a specific CXCR4 inhibitor, was also used in CXCL12-treated cells. Treatment with AMD3100 produced a reduction in tube formation in HUVECs. Thus, it can be concluded that CXCL12-CXCR4 promotes tube formation in HUVECs.

Regarding the biological functions of CXCL12 and CXCR7 in cells, existing reports have focused on cell proliferation^47^, invasion^48^ and migration^49^. To date, the role of CXCR7 in regulating angiogenesis in HUVECs has remained unclear. In our exploration of effects of the CXCL12-CXCR7 axis and its precise mechanisms in HUVECs, we observed that CXCL12 treatment enhanced angiogenesis in the CAM model and in HUVECs. We also observed significantly increased cell migration and tube formation after treatment with CXCL12 or TC14012, a CXCR7 agonist. Our study thus indicated the significance of CXCR7 activation on tube formation in HUVECs. These results are consistent with recent studies that showed that CXCR7 induced the angiogenic capacity of tumor cells^24, 48^.

Our recent studies have demonstrated that CXCL12 activated CXCR7 for stem cell polarization, migration and prevention of apoptosis^50, 51^. Those studies implied that CXCR7 acts through an arrestin-2-mediated pathway, independently of CXCR4, and established a single-cell-based polarization and migration model^50^. Clarifying whether CXCR7 affects polarization and migration of ECs and the formation of new vessels is worthwhile, since vessel sprouting demands migration and polarization of ECs in response to cytokines^3^. Our studies have shown that both CXCR7 and CXCR4 may be involved in the polarization of HUVECs in the stripe assay, which is consistent with the migration data. These results led us to investigate the possibility that the regulation of EC polarity via CXCR7 signaling mediates other molecular targets; however, it is not yet known how CXCR7 regulates the polarity of HUVECs or how it induces their directional migration.

To verify the angiogenic roles of CXCR7 *in vivo*, we utilized the Matrigel plug model. We found that both TC14012 and CXCL12 could increase the hemoglobin content and the number of vessels in the Matrigel plugs. These *in vivo* results demonstrated the angiogenesis-promoting effects of CXCR7, supporting CXCR7 as an important modulator of the CXCL12- or TC14012-mediated regulation of angiogenesis. In our study, we did not investigate the molecular mechanisms by which CXCR7 stimulated angiogenesis *in vivo*.

Recent reports have demonstrated that CXCR7 can induce phosphorylation of Akt in many cell lines^52–54^. Our data implied that the PI3K/Akt signaling pathway was necessary for the initiation of angiogenesis by CXCR7. Another recent study of ours suggested that the CXCR7-mediated signaling pathways are independent of those activated through CXCR4^51^. Thus, further studies elucidating the molecular mechanism of the signaling pathways activated by the CXCL12/CXCR7 axis are required.

In conclusion, the angiogenic effects of the CXCL12/CXCR7 axis on HUVECs may act through the PI3K/Akt signaling pathway, inducing cell proliferation, migration, and tube formation. In addition, CXCL12 induced directional polarization of CXCR7 expression and of HUVECs. Furthermore, the *in vivo* results suggested that CXCR7 could significantly induce blood vessel growth. Our data confirmed that the CXCL12-CXCR7 axis accelerates angiogenesis of HUVECs through Akt signaling. Thus, this axis might function as a potential therapeutic target for angiogenesis. Although our study demonstrated key roles for CXCR7 in the migration and angiogenesis of HUVECs, the roles of the CXCL12/CXCR7 interactions in inflammatory or stress microenvironments remain unknown. Thus, further studies elucidating the role of the CXCL12/CXCR7 axis in inflammatory or stress environments are needed.

## Materials and Methods

### Cell culture

Human umbilical cord vein endothelial cells (HUVECs) (AllCells, Shanghai, China) were cultured for up to 5–7 passages in complete endothelial cell growth medium (H004, AllCells, Shanghai, China) (unless otherwise specified). Complete endothelial cell growth medium consists of 500 mL of basal medium, 5% (v/v) fetal bovine serum, 1% (v/v) endothelial cell growth supplement and 1% (v/v) penicillin/streptomycin solution. The cells were maintained at 37 °C in a humidified 5% CO_2_ incubator.

### Antibodies and reagents

Recombinant human CXCL12 was obtained from R&D Systems (Wiesbaden, Germany). Calcein-AM was purchased from Dojindo (Dojindo Laboratories, China). Polyclonal rabbit anti-CXCR4 and polyclonal rabbit anti-CXCR7 were obtained from Abcam (Cambridge, MA, USA). Polyclonal rabbit anti-β-Actin antibody, TC14012 and AMD3100, were obtained from Sigma (Deisenhofen, Germany). CCX771 was obtained from ChemoCentryx (Mountain View, CA, USA).

### Chicken chorioallantoic membrane (CAM) assay

To evaluate the effect on angiogenesis, the CAM assay was performed using fertilized chicken eggs on the 8th day of development. A 1-cm-diameter window was opened in the shell of each egg containing an 8-day-old chicken embryo (Yunyi Breeding Co. Ltd, Guangdong, China). The surface of the dermic sheet on the floor of the air sac was removed to expose the CAM. A 0.5-cm-diameter gelatin sponge was first placed on top of the CAM, and CXCL12 was added to the center of the gelatin sponge. After the window was closed with sterile adhesive tape, the eggs were incubated at 37 °C under 80–90% relative humidity for 48 h. Following fixation with the stop solution (methanol:acetone for 15 min, the CAMs were cut and harvested, and gross photos of each CAM were taken with a digital camera (Carl Zeiss, Jena, Germany) at 10× and 40×. The amount of angiogenesis was quantified using ImageJ v1.50b software (National Institutes of Health, Bethesda, MD).

### HUVEC proliferation assay

HUVECs (2 × 10^4^) were suspended in Dulbecco’s Modified Eagle Medium (DMEM) containing 0.5% FBS and seeded into 24-well plates. Recombinant human CXCL12 (0–100 ng/mL) was added to the cells in the presence or absence of pre-treatment with either the CXCR4 antagonist AMD3100 or the CXCR7 antagonist CCX771. The antagonist was added 30 min prior to CXCL12 treatment. Three days later, the living cells were stained with 2 μM calcein-AM, and the total cell numbers were counted in three independently processed wells each time using a digital microscope (Carl Zeiss, Jena, Germany) with wavelengths set to an absorbance maximum of 493 nm and an emission maximum of 514 nm. For each condition, three independent experiments were performed, and the mean numbers of proliferating cells are reported.

### Transwell cell migration assay

Cell migration was performed using Transwell inserts in 24-well culture dishes (polycarbonate membrane insert of 6.5 mm in diameter and with 8.0 μm pores; Corning, Schiphol, Netherlands). HUVECs (2 × 10^4^) were suspended in DMEM containing 0.5% serum and added to the upper chamber. CXCL12 (0–100 ng/mL) was added into the lower chamber. In other experiments, the CXCR4 antagonist AMD3100 or the CXCR7 antagonist CCX771 was first added to the HUVECs. The antagonist was added 30 min prior to CXCL12 treatment. Twenty-four hours later, the cells on the upper surface of the membrane were removed by gentle scraping with a cotton swab. The cells on the lower surface of the membrane were stained with 2 μM calcein-AM for 30 min. The migrated cells were randomly counted in eight microscopic fields, and the numbers of cells were quantified by using Image-Pro Plus 6.0.

### Cell tube formation assay

HUVECs (2 × 10^4^) were suspended in DMEM containing 0.5% serum and seeded into Matrigel-coated wells of a 96-well plate, and then CXCL12 (0–100 ng/mL) was added. In other experiments, the CXCR4 antagonist AMD3100 or the CXCR7 antagonist CCX771 was first added to the HUVECs. After twenty-four hours of incubation in an incubator (5% CO_2_), photographs were taken at low magnification (5X) with a digital microscope (Carl Zeiss, Jena, Germany), and the tubes were counted. Only perfectly continuous tubes between two branching points were evaluated. For each condition, three independent experiments were performed, and the mean tube numbers are presented.

### Matrigel plug assay

Matrigel plug assay was used to detect angiogenesis *in vivo* ^55^. Matrigel (500 μL) containing either TC14012 (30 μM) or CXCL12 (100 ng/mL) were inoculated subcutaneously into the right flank of Balb/c mice (6 to 8 week old). The mice were housed at 23 °C on a 12 h light/12 h dark cycle with food and water supplied ad libitum at Tongji University. Negative controls were obtained by injecting mice with Matrigel alone. Each group consisted of four mice. After 10 days, the Matrigel plugs were removed, and the hemoglobin content was determined according to Drabkin’s method. The relative hemoglobin content was calculated against the negative controls. The plugs were fixed with 4% paraformaldehyde in PBS for 2 h at room temperature and then transferred to 70% ethanol, embedded in paraffin, and processed for hematoxylin and eosin (H&E) staining. The formation of new microvessels was counted in four fields of view using a 20× objective lens.

### Stripe assay

The stripe assay model was used in a similar way as indicated by a previous report^50^. Polydimethylsiloxane casted on a silicon base created micro channels (100 × 100 μm), and the channels were coated with fibronectin and poly-D-lysine. Fluorescein conjugated albumin (from BSA) or CXCL12 (100 mg/mL) was added to the microchannels, and a vacuum was used on the other side of the micro channels. The polydimethylsiloxane molds were removed for 6 h, and the stripes were used. The HUVECs were grown on cover glasses precoated with the micro stripes of BSA/BSA-CXCL12 to study cell polarization. The cells were then stained with antibodies against CXCR4 (R&D Systems, MAB21651, 1:200), CXCR7 (R&D Systems, MAB42273, 1:100) and F-Actin (coupled with Rhodamine, R415, Life Technologies). Images were obtained with a confocal laser-scanning microscope and quantified by using Image Pro Plus.

### Western blot

HUVECs were seeded in 6-well plates and exposed to CXCL12 (0–100 ng/mL). The protein concentration of the supernatants was determined by using the BCA protein assay (Beyotime, China). The cells were collected at the indicated time points, and whole cell lysates were prepared in RIPA solution. Total lysate proteins (50 μg) were resolved by 10% sodium dodecyl sulfate-polyacrylamide gel electrophoresis (SDS-PAGE) and blotted onto polyvinylidene fluoride (PVDF) membranes. After the transfer, the membranes were blocked with 5% skimmed milk in 0.1% Tween-20 Tris-buffered saline (TBST) for 60 min. Then, the membranes were incubated with antibodies against CXCR4, CXCR7 (Abcam), Akt and pAkt overnight at 4 °C. The membranes were probed with a horseradish peroxidase-conjugated rabbit anti-goat or goat anti-rabbit secondary antibody for 1 h at room temperature. The antibodies were detected with an enhanced chemiluminescence (ECL) kit (GE Healthcare, Munich, Germany). At least three independent Western blots were performed and densitometrically analyzed for each experiment.

### Statistical analysis

Data are expressed as the mean ± standard deviation (S.D.). Comparisons of more than two groups were performed using one-way ANOVA followed by Tukey’s post hoc tests. Comparisons between two groups were generated with two-tailed t-tests. Statistical analyses were performed using SPSS 10.0 for Windows. *P* < 0.05 was considered statistically significant.

### Data availability

Data supporting the findings of this work are available within the article and from the corresponding author on reasonable request.
